# Effects of Omega 3 Fatty Acids on Main Dimensions of Psychopathology

**DOI:** 10.3390/ijms21176042

**Published:** 2020-08-21

**Authors:** Paola Bozzatello, Maria Laura De Rosa, Paola Rocca, Silvio Bellino

**Affiliations:** 1 Department of Neuroscience, Faculty of Medicine, University of Turin, 10126 Turin, Italy; paola.bozzatello@unito.it (P.B.); marialauraderosa4@gmail.com (M.L.D.R.); paola.rocca@unito.it (P.R.); 2 Center for Personality Disorders, Psychiatric Clinic, 10126 Turin, Italy

**Keywords:** omega-3 fatty acids, PUFAs, psychiatric disorders, psychosis, affectivity, impulsivity, self-harm behaviors

## Abstract

The usefulness of polyunsaturated fatty acids on inflammatory, cardiovascular, and the nervous system was studied in the last decades, but the mechanisms underlying their benefic properties are still partially unknown. These agents seem to express their action on the membrane phospholipid composition and permeability and modulation of second messenger cascades. In psychiatry, the efficacy and tolerability of omega-3 fatty acids were investigated in several psychiatric disorders, including major depression, bipolar disorder, personality disorders, high-risk conditions to develop psychosis, attention-deficit hyperactivity disorder, and autism spectrum disorders. Initial findings in this field are promising, and some relevant questions need to be addressed. In particular, the effects of these agents on the main symptom dimensions have to be investigated in a trans-diagnostic perspective. The present systematic review is aimed to examine the available data on the efficacy of omega-3 fatty acids on domains of psychotic symptoms, affective symptoms, impulsivity, and aggressiveness, and harmful behaviors, and suicide risk.

## 1. Introduction

In the last decades, the scientific literature has highlighted an urgent need to identify new compounds to treat psychiatric disorders in order to improve and boost available medications that entail not insignificant side effects. [1] As the harmful effects of the western diet, the absence of polyunsaturated fatty acids (PUFAs), on immune memory and inflammation processes are recognized [2], several studies evaluated the relationships between the low plasma level of long-chain polyunsaturated fatty acids and several medical conditions. [3] The effects of omega-3 fatty acids (n-3 PUFAs), including α-linolenic acid (ALA), eicosapentaenoic acid (EPA), and docosahexaenoic acid (DHA), and of omega-6 fatty acids (n-6 PUFAs), including linoleic and arachidonic acid on overall human health have been extensively studied [4,5,6].

Nowadays, a consistent body of literature supports the importance of polyunsaturated fatty acids in brain function and the effect of their supplementation in mental disorders, but the molecular mechanisms underpinning their action are not completely clear [7,8,9]. Omega-3 fatty acids are essential fatty acids that humans and other animals require for good health, but cannot be synthesized. So, these compounds are introduced into the organism with food and metabolic conversion. The lipid derivatives of omega-3 PUFAs include anti-inflammatory eicosanoids, such as resins and resolvins, which mediate the effects on immune function [10,11]. Long-chain PUFAs, including at least 20 carbon atoms, have important functional roles as components of phospholipids forming the cell membranes [12], and as signaling molecules in all tissues, including the brain [13,14]. DHA is an essential omega-3 long-chain polyunsaturated fatty acid involved in many cellular processes in mammalian cells [15], represents approximately 60% of PUFAs in neuronal membranes [16], and is a precursor for bioactive compounds that modulate cell signaling and gene expression [17]. It takes part in neuronal growth, development, and function, acting as a neurotrophic factor and modulating synaptic activity [18], and it has been found to reduce the deleterious effects of chronic activation of inflammatory signaling cascades in the brain and to ameliorate neurotoxic injuries implicated in neurodegenerative disorders [19].

Some investigations showed that cellular molecules such as resolvins, protectins, peroxisome proliferator-activated receptor γ (PPAR-γ), AMP-activated protein kinase (AMPK), and nuclear factor kappa-light-chain-enhancer of activated B cells (NF-κB) are potential targets of the anti-inflammatory action of DHA [20,21,22]. Other authors observed that DHA supplementation in an aged rat model could reduce cerebral reactive oxygen species, prevent age-related cognitive dysfunction [23], and decrease oxidative stress in rats subjected to permanent cerebral ischemia [20]. Another potential mechanism of the action of DHA is the regulation of the retinoid X receptor (RXR) that is a crucial developmental and survival factor implicated in multiple transcription pathways, such as the dopaminergic system [24].

Supplementation with uridine, DHA, and choline resulted in enhanced levels of phosphatidylcholine (PC), as well as other membrane phospholipids in the brains of healthy adult rodents [25]. The same effect was observed when DHA was substituted with EPA, but not with the omega-6 PUFA arachidonic acid (AA) [26]. The combination of DHA and EPA also intensified membrane phospholipid levels in brains of pups born from rats supplemented during gestation and nursing and produced an increased number of dendritic spines per unit area and the improvement of synaptic activities [25,27]. These findings suggested that synaptogenesis is partially determined by the availability of phospholipid precursors into the brain. In fact, phospholipid precursors supplementation in animal models produced biochemical and structural modifications promoting the release of neurotransmitters, such as dopamine [28] and acetylcholine [29], and improvement in specific cognitive tasks [30].

Supplementation with phospholipid precursors not only enhanced synaptogenesis in normal conditions, but also provided benefit under degenerative conditions that are characterized by synaptic deficiency both in laboratory rodents and humans. The restorative effects of supplementation with uridine monophosphate (UMP), DHA, and choline were indicated by increasing striatal dopamine levels and tyrosine hydroxylase (TH) activity, and by enhancing levels of membrane phospholipids and synaptic proteins in a rat model of Parkinson’s disease [31].

On the basis of these preclinical findings [32], several studies have been performed to test the usefulness of these agents in clinical conditions. Recent reviews [7,8,9,10] and meta-analyses [33,34] supported the role of PUFAs in the treatment of psychiatric disorders. In particular, the efficacy and tolerability of omega-3 fatty acids (DHA and EPA) were investigated in major depression, bipolar disorder, personality disorders, high-risk conditions to develop psychosis, attention-deficit hyperactivity disorder, and autism spectrum disorders. An interesting perspective is to deal with the effects of these agents in a trans-diagnostic perspective and investigate their effects on the main psychiatric symptom dimensions. The present systematic review is aimed to examine available data on the efficacy of omega-3 fatty acids on domains of psychotic symptoms, affective symptoms, impulsivity, and harmful behaviors.

## 2. Psychotic Symptoms

Deficits in long-chain n-3 PUFAs are one of the mechanisms thought to contribute to the risk of psychosis [35]. In schizophrenia and other psychotic disorders, the phospholipid structure of the cell membrane is altered and these modifications may contribute to various features of the pathophysiology observed in psychotic disorders, including neurotransmission, immune activation, and antioxidative defense [36]. This hypothesis is supported by studies that showed a plasma n-3 PUFAs deficiency in patients with schizophrenia compared to controls. [37] Besides, some evidence has suggested that the onset of psychotic disorders arises during human brain development in which n-3 PUFAs play a central role [38]. So, n-3 PUFAs supplementation could have an effect in preventing transition to psychosis [39,40], in particular, in high-risk populations [41].

These initial promising findings allow us to take into consideration the use of non-pharmacological compounds for early interventions in young people at risk for psychosis [42]. The results obtained so far about PUFAs as an add-on strategy in the treatment of schizophrenia are controversial; while several studies [40,41,42,43,44,45,46,47,48,49,50,51,52,53,54,55,56,57] produced favorable data, some others [58,59,60,61] showed no significant differences in clinical outcomes.

The symptoms dimension of psychosis is characterized by abnormalities in five symptom domains: delusions, hallucinations, disorganized thinking, disorganized behaviors, and negative symptoms. Lower levels of n-3 PUFAs seem to correlate with more severe negative symptoms in ultra-high risk (UHR) patients for psychosis and could be adopted as a biomarker that predicts the conversion to psychosis in UHR subjects [43].

Some findings suggested a role of EPA as an add-on therapy (in combination with antipsychotic therapy) in general, positive, negative, and depressive symptoms [46,47,49,50,51] in patients with UHR and first episode of psychosis. EPA or DHA augmentation to antipsychotics reduced the deterioration of hippocampus tissues with a positive effect on negative symptoms [47], decreased the oxidative stress status of plasma with a positive effect on global and negative symptoms [50], and increased the telomerase levels in peripheral blood cells with a positive effect on the severity of illness [51].

Some authors suggested that in stable schizophrenia, omega-3 fatty acid supplementation had a beneficial effect on positive symptoms (delusions and hallucinations) [52,55]. Among PUFAs, EPA has been found superior than placebo and also than DHA in reducing positive [52,55] and negative symptoms [54]. Moreover, supplementation with EPA induced a less severe impairment of the course of psychosis [56].

Previous findings [62] showed that omega-3 fatty acids have beneficial effects on triglycerides in patients with psychotic symptoms and metabolic syndrome and may enhance the brain-derived neurotrophic factor (BDNF) levels through their anti-inflammatory properties with a reduction of cognitive dysfunction in these patients [63]. Probably, an appropriate dietary supplementation could play a partially therapeutic effect even in more severe patients, improving some behavioral aspects and reducing the cognitive deterioration [64]. In addition, EPA supplementation was found to be associated with a marked increase of glutathione, an antioxidant agent, in patients with first episode of psychosis. [46]

Further studies are needed to investigate the role of omega-3 fatty acids as a treatment for the specific clusters of psychotic symptoms in psychiatric disorders other than schizophrenia. In fact, there is lack of evidence regarding the efficacy of PUFAs on the psychotic cluster of symptoms in bipolar disorder, schizoaffective disorder, and schizotypal personality disorder. To our knowledge only one case report documented the benefits of omega-3 fatty acids supplementation (EPA 1.187 g/day + DHA 0.613 g/day) in association with cholecalciferol (vitamin D3) and mood stabilizers for psychotic features in bipolar disorder [65].

In apparent contrast with the expectations, higher scores on positive schizotypal trait measures in healthy adults were found to be associated with higher concentrations of omega-6 and omega-3 fatty acids in red cell membranes [66]. These findings seem to support the hypothesis that high blood levels of PUFAs may confer some protection against psychotic breakdown [67].

Main findings are displayed in Table 1.

## 3. Affective Symptoms

Affective symptoms include depression, hypomania, mania, and anxiety symptoms. Abnormalities in affectivity characterize mood disorders but are also frequently associated with other mental disorders, such as psychotic and personality disorders. The role of PUFAs in treating affective symptoms such as depression can be linked to the inflammation hypothesis of the pathogenesis of depression and to the effects of EPA and DHA in reducing the effects of chronic activation of the inflammatory cascade [68]. In addition, it has been found that patients with major depressive disorder (MDD) have a lower level of EPA and DHA in their peripheral tissues (plasma, serum, and red blood cells) than control subjects [69].

The antidepressant efficacy of PUFAs could be partly explained by their role in myelinization processes—several models have been proposed to explain oligodendroglia cell loss/dysfunction in MDD. Increased levels of circulating corticosterone linked to the over-activation of the hypothalamic, pituitary, adrenal (HPA) axis in stress conditions could be at the origin of oligodendroglia loss/dysfunction in depression [70]. Studies in rats show that lower omega-3 PUFAs intake causes abnormalities of myelin [71] and that omega-3 PUFAs administration stimulates the expression of myelin proteins through the activation of signaling pathways involved in brain development [72]. Some experimental brain injury studies in rodents support this association and conclude that the supplementation with n-3 PUFAs increases the degree of differentiation of oligodendroglia cells [73,74].

Furthermore, an unbalanced omega-3/omega-6 ratio could lead to a range of functional consequences in the monoamine transport system [75]. In fact, there is evidence that severe n-3 PUFA deficiency alters the dopaminergic and serotonergic transmission systems, inducing an increased vulnerability to mood disorders. The mesolimbic dopaminergic pathway was more active, whereas the mesocortical pathway was found to be less active in rats with n-3 PUFA deficiency. This imbalance in dopaminergic neurotransmission could be related to symptoms of depressive episodes, such as lack of motivation, decreased response to reward, and impairment in intellective abilities [76].

The inflammatory response and the stimulation of BDNF have been hypothesized as two potential mechanisms through which omega-3 fatty acids could provide their anxiolytic effect. In particular, as anxiety is associated with an increased production of pro-inflammatory cytokines, including tumor necrosis factor-alpha (TNF-α) and interleukin-6 (IL-6), the effect of n-3 PUFAs of decreasing inflammatory cytokines indirectly promote the reduction of anxious symptoms. Moreover, when BDNF is low, it fails to stimulate synaptic growth of serotonergic neurons in the brain, and low levels of serotonin are associated with anxiety. Thus, BDNF could reduce depression and anxiety by stimulating synaptic growth of serotonergic neurons in the brain [77,78].

A consistent number of studies [79,80,81,82,83,84,85,86,87,88,89,90,91,92,93,94,95,96,97,98,99,100,101,102,103,104,105,106,107,108,109,110,111,112] investigated the role of PUFAs in the treatment of major depressive disorder (MDD). Although some of them [79,80,81,82,83,84,85,86,87,88,89,90,91,92,93,94,95,96] found no significant data, several trials [97,98,99,100,101,102,103,104,105,106,107,108,109,110,111,112] showed an improvement of depressive symptoms in patients treated with n-3 PUFAs in combination with antidepressants. In particular, a combination of higher dose of EPA with lower dose of DHA (EPA 1.67 g/day + DHA 0.83 g/day), added to existing antidepressant treatment, revealed beneficial effects on mood [102,103]. On the contrary, lower doses of EPA with higher doses of DHA (EPA 0.6 g/day + DHA 2.2 g/day added to standard therapy) evidenced no benefits on depressive symptoms [85]. Some authors [108] stated that higher doses of n-3 PUFAs (EPA 2.1/day + DHA 2.5 g/day) are more efficacious on depressive symptoms [85,86]. Several studies [113,114,115,116,117,118,119,120] investigating the efficacy of n-3 PUFAs supplementation (2.5 g/day with EPA:DHA = 2:1) in patients who suffer from bipolar disorder treated with mood stabilizers confirmed the effect of these agents on depressive symptoms [120] but not on manic ones [8,9]. Unlike major depression, in the case of bipolar disorder, higher doses of EPA (4.4 g/day) and DHA (2.4 g/day) seem to be not useful to obtain a better improvement of affective symptoms [113]. The reviews [121,122,123,124] and meta-analyses [33,125] on this topic concluded that there is a pressing need for further investigations, especially concerning the manic symptoms.

With regard to anxious symptoms, observational and experimental studies have demonstrated that ω-3 supplementation could ameliorate anxiety symptoms. High ω-3 fatty acid intake is associated with reduced likelihood of meeting the diagnostic criteria for anxiety disorders [126,127] and lower levels of self-reported anxiety symptom severity [128,129]. However, the literature data on the efficacy of PUFAs on anxiety are still scarce and inconclusive. Two trials [130,131] suggested a positive effect of n-3 PUFAs (EPA 2.25 g/day + DHA 0.5 g/day) on anxiety in patients with substance abuse and one open-label study reported an improvement of anxious symptoms in patients with post-traumatic stress disorder [132].

Depressive and anxious symptoms often occur in patients with schizophrenia or other psychotic disorders. In particular, young people at UHR to develop psychosis who present affective symptoms (depression and anxiety) show lower plasma levels of omega-3 fatty acids and higher levels of omega-6 fatty acids then controls [41]. On this basis, some authors investigated the effects of EPA and DHA supplementation in patients with schizophrenia and depressive and anxious symptoms. The results showed that EPA at doses ranging between 1 and 4 g/day is efficacious in reducing the depressive symptoms [50,54] and anxious symptoms [54] of schizophrenia. Among personality disorders, n-3 PUFAs were found efficacious on depressive symptoms of patients with borderline personality disorder (BPD), both in monotherapy (EPA 1 g/day) [133] and in addition to standard pharmacotherapies (EPA 1.2 g/day + DHA 0.9 g/day) [134]. 

Main findings are displayed in Table 2.

## 4. Impulsive and Aggressive Symptoms

There is cross-sectional evidence that deficiency of omega-3 fatty acids is associated with hostility, aggressive behaviors, and impulsivity in both psychiatric and non-psychiatric populations [135]. The relationship between omega-3 PUFAs and aggression have also been explored in rodent studies, that have found an increase of aggressive behaviors with both omega-3 PUFAs deficiency [136] and high-omega-6 PUFAs intake [137]. Omega-3 administration seems to have beneficial effects in reducing aggression among the general population [138]. A study performed in a group of Australian inmates found that the n-3 PUFAs levels were inversely related to the degree of aggressive behavior and hostility [139]. The results of several studies collected in a meta-analysis [140] are encouraging for the use of omega-3 fatty acid supplementation to prevent and reduce aggressive behaviors in both children and adults. The relationship between abnormal PUFAs distribution and conditions characterized by a high-level of impulsivity could be partly explained by the effect of these compounds on the serotonin system and membrane stability. In particular, EPA influences serotonin release and DHA improves membrane-embedded serotonin receptor accessibility with an increase of cognitive function, propensity for prosocial behavior, and of impulsive behavior control [141].

Higher omega-6/omega-3 ratios have been found in subjects with anger, irritability, and aggressiveness, in particular during exposure to elevated inflammatory cytokines [142]. Thus, inflammation and inflammation-related genetic polymorphisms are two possible additional sources of variability that may contribute to understanding the biological substrates of aggressive states [143].

The relationship between omega-3 PUFAs and aggressive and impulsive behaviors have been observed in various psychiatric contexts. It is possible that omega-3 fatty acids may alter brain functionality prior to emergent or detectable behavioral changes [135].

Lower plasma levels of total omega-3 PUFAs and a trend toward a higher ratio of omega-6 to omega-3 PUFAs were observed in conditions with a high level of impulsivity. For example, among subjects with gambling disorder, a higher percentage composition of EPA and a lower AA/EPA ratio and AA/DHA ratio in the red blood cell membrane was observed in impulsive gamblers, compared with non-impulsive ones [144]. In a similar way, cocaine abusers with impulsive and aggressive behaviors showed an imbalance between omega-3 and omega-6 fatty acids [145,146], and violent males and impulsive offenders hospitalized in a forensic psychiatric unit presented a lower plasmatic DHA level than controls [147].

Some available evidence suggests a beneficial effect of omega-3 supplementation on aggressive and antisocial behavior in adolescence and adulthood [148]. The mechanisms underpinning these results is still unclear, although the upregulation of dysfunctional prefrontal regions is one candidate mediator [149,150].

Studies that evaluated the effects of PUFAs on impulsivity and aggressiveness in major psychiatric disorders indicated that the addition of rather low doses of omega-3 fatty acids (EPA 0.54 g/day + DHA 0.36 g/day) to antipsychotic treatment might reduce agitation and violent behaviors in inpatients with schizophrenia in the chronic phase [61]. Single therapy with omega -3 fatty acids (EPA 0.93 g/day + DHA 0.29 g/day) showed an improvement of impulsive dyscontrol and aggressiveness in patients affected by ADHD (attention deficit hyperactivity disorder) [151,152] and in patients affected by BPD [133,134]. Moreover, combined therapy with EPA (1–1.2 g/day) plus DHA (0.6–0.9 g/day) and valproic acid (800–1300 mg/day—plasma range: 50–100 µg/mL) was superior to single therapy with valproic acid on impulsive–behavioral dyscontrol and outbursts of anger in BPD patients [153]. Combined therapy with omega-3 fatty acids showed long-lasting effects at the end of 24 weeks of follow-up in terms of anger control [154]. 

Main findings are displayed in Table 3.

## 5. Self-harm Behaviors and Suicidal Conducts

Self-harm behaviors include self-injuries, such as cutting and self-mutilation, burning, scratching, or hitting body parts [155]. The desire to self-harm is a common symptom of some personality disorders, but is frequent also in patients with mood disorders, anxiety disorders, substance use disorders, eating disorders, post-traumatic stress disorder, and schizophrenia [156]. The classification challenge presented by repetitive, non-lethal, self-harm is reflected in the numerous terms used for it in the historical literature, including self-mutilation, focal suicide, parasuicide, suicide gesture, wrist-cutting syndrome, delicate self-cutting, deliberate self-harm, self-injury, and self-injurious behavior. Currently, DSM-5 [157] includes several types of repetitive, non-lethal self-injurious behaviors: nonsuicidal self-injury (NSSI), trichotillomania (hair-pulling disorder), excoriation (skin-picking) disorder, and suicidal behavior disorder.

A theoretical model indicates the pathways whereby cholesterol-lowering treatments could affect PUFAs and lipid rafts, leading to alterations in serotonergic neurotransmission, corticotrophic function, and inflammation, and thereby increase parasuicidal and suicidal risk. So, there could be a preventative intervention, with respect to suicide and parasuicide, in administering n-3 PUFA supplements to patients for whom lower cholesterol is medically important and who have or develop psychiatric vulnerabilities, for example, patients with cardiac diseases or metabolic syndrome [158,159].

Unfortunately, studies on the effect of PUFAs on self-harm behaviors and suicide are still sparse. Some of these were performed in subjects without a psychiatric diagnosis and showed that low n-3 PUFAs plasma levels were associated with an increased number of deaths for suicide compared with other causes of death in active duty US military personnel. Moreover, veterans treated with PUFAs reported a reduction in suicidal ideation after omega-3 fatty acid supplementation [160,161].

Other investigations found that low EPA and DHA percentages, elevated omega-6/omega-3 ratio, and higher AA concentration seem to be associated with increased self-harm and suicidal attempts in patients with mood disorders [162,163,164,165]. Having low levels of arachidonic acid and receiving a combination of high dose of EPA and lithium were found protective for suicide attempts and deliberate self-harm in the case of bipolar disorder [166].

In BPD patients, n-3 PUFAs supplementation (EPA 1–1.2 g/day + DHA 0.6–0.9 g/day), added to standard psychiatric therapies, showed a significant reduction of self-harming and parasuicidal attitudes [133,134,153,154]. In particular, the reduction of suicidal behaviors seems to be independent of change in the depression score [134]. 

Main findings are displayed in Table 4.

## 6. Conclusive Remarks

Recent evidence supports the importance of polyunsaturated fatty acids in brain functioning and the action of their supplementation in psychiatric disorders, but the underlying mechanisms of the potential preventive and therapeutic effect of PUFAs is still unclear.

Preclinical studies hypothesized that omega-3 fatty acids may attenuate stress-related changes in animals with depressive features, as well as in humans [167]. These agents seem to be involved in myelinization processes and synaptic pruning, that are fundamental processes during brain development. PUFAs have immune-modulatory, and anti-inflammatory properties through the modulation of omega-6 fatty acids and the promotion of resolvins, neuroprotective factors, and anti-inflammatory mediators. They are also involved in membrane fluidity producing an improvement of monoaminergic transmission.

In the last decades, the role of long-chain PUFAs in the treatment of several psychiatric disorders has gradually increased, as confirmed by the growing number of randomized controlled trials testing the efficacy of essential fatty acids, especially omega-3 supplementation. Nevertheless, an overall agreement about their efficacy is still lacking, and the results of most trials are controversial and inconclusive. Differences in methods, including sample size, selection criteria, choice, and dosage of fatty acids (i.e., EPA, or DHA, or a combination of the two), and the duration of supplementation, often make results not comparable.

The aim of this review is to evaluate, in a trans-diagnostic perspective, the efficacy of omega-3 fatty acids on the main psychiatric symptom dimensions, in particular on domains of psychotic symptoms, affective symptoms, impulsivity, and harmful behaviors.

Concerning psychosis, the available data on the effects of EPA and DHA needs to be replicated. However, initial findings are rather promising and show that these compounds, in addition to antipsychotics, may improve all symptom domains of psychosis, in particular negative symptoms. Evidence on the effects of PUFAs as single therapy for schizophrenic patients and on psychotic symptoms that are part of the clinical picture of psychiatric disorders other than schizophrenia are not sufficient.

The available literature reported promising data also on the role of n-3 PUFAs in the treatment of affective symptoms. In particular, these compounds, administered at high doses (1–2 g/day, with a ratio EPA:DHA = 2:1), seem able to improve depressive symptoms, both in monotherapy and in addition to antidepressants in major depression, in combination with mood stabilizers in bipolar depression, in addition to antipsychotics in schizophrenia. Evidence of the efficacy of manic symptoms is lacking, while the effects on anxiety need to be clarified and confirmed.

Concerning impulsivity and aggressiveness, the initial results on the beneficial effects of EPA and DHA supplementation are encouraging, both in single and combined interventions, and induce a decrease in the levels of impulsivity, outbursts of anger, and overt aggression. This area of psychopathology needs to be explored in depth by future investigations to allow us to draw more reliable conclusions. Another symptom domain that appears to be sensitive to the action of PUFAs is that of self-harm conduct. The effects of PUFAs were studied in patients with a high risk of harmful and suicidal behaviors, mainly in subjects with severe personality disorders, but also in patients with mood disorders. Additional important factors that require evaluation in this population include lipid-associated genetic variants and epigenetic markers that modulate the effects of lipid status and of PUFAs supplementation on self-harm and suicide risk.

In conclusion, polyunsaturated fatty acids are dietary supplements that can have a role on the basis of initial research data in treating several symptoms of psychiatric disorders, at least in combination with traditional medications. The lack of severe adverse effects is a significant reason to carefully consider the therapeutic potential of these agents. However, we have no sufficient evidence to draw final conclusions and to propose official indications for PUFAs in psychiatry. This is mainly due to serious limitations and considerable heterogeneity in the design and methods of available trials. As is suggested by the organization of this review, the authors’ opinion is that less controversial and discordant data could be obtained in incoming studies by focusing, not on specific categorical diagnoses, but on psychopathological dimensions and symptom domains observed from a trans-nosographical perspective. Such dimensions could be the direct expression of the pathophysiological and biochemical pathways on which PUFAs actually produce their beneficial effects.

## Figures and Tables

**Table 1:** Randomized controlled trials and open trials of omega-3 PUFAs on psychotic symptoms.

Study (Year) [Ref.]	Interventional Arm(s)	Comparison Arm(s)	Sample	Treatment Duration	Results
Amminger et al., 2013 [40]	EPA 700 mg/day + DHA 480 mg/day + 7.6 mg vitamin E	placebo	81 young individuals at UHR	12 weeks	↓ positive symptoms, negative symptoms and general symptoms, ↑ level of functioning
Amminger et al., 2015 [42]	EPA 700 mg/day + DHA 480 mg/day	placebo	81 young individuals at UHR	6.7 years follow-up	↓ risk of progression to psychotic disorder and psychiatric morbidity
Amminger et al., 2010 [43]	EPA 700 mg/day + DHA 480 mg/day	Placebo	81 individuals at UHR	12 weeks	↓ progression in psychosis in young UHR patients
Smesny et al., 2014 [44]	EPA 700 mg/day + DHA 480 mg/day	Placebo	81 young individuals at UHR	12 weeks	normalizing PLA2 activity and d-6-desaturase-mediated metabolism of o-3 and o-6 PUFAs
Berger et al., 2007 [45]	Ethyl-EPA 3 g/day + antipsychotics	Placebo + antipsychotics	69 patients at FEP	12 weeks	accelerated treatment response
Berger et al., 2008 [46]	EPA 2 g/day + Antipsychotics	Placebo + antipsychotics	24 patients at FEP	12 weeks	↓ of negative symptoms
Wood et al., 2010 [47]	Ethyl-EPA 2 g/day + antipsychotics	Placebo + antipsychotics	17 patients at FEP	12 weeks	increased water in hippocampal tissues and positive effect on negative symptoms
Emsley et al., 2014 [48]	EPA 2 g/day + DHA 1 g/day + α-LA 300 mg/day	placebo	33 patients after FEP on antipsychotic discontinuation	2 years	relapse prevention of psychotic symptoms
Pawelzcyk et al., 2016 [49]	EPA + DHA 2.2 g/day	Placebo	71 patients at FEP	26 weeks	↓ psychotic symptoms measured with PANSS ↓ depressive symptoms ↑ level of functioning
Pawelzcyk et al., 2017 [50]	EPA + DHA 2.2 g/day	placebo	71 patients at FEP	26 weeks	improved PANSS negative and general symptoms, along with global functioning
Peet et al.,2001 [52]	EPA or DHA 2 g/day + antipsychotics	Placebo + Antipsychotics	45 patients with stable schizophrenia	12 weeks	↓ psychotic symptoms measured with PANSS in the group treated with EPA
Peet et al., 2002 [53]	Ethyl-EPA 1–4 g/day + antipsychotics	Placebo + antipsychotics	115 patients with stable schizophrenia	12 weeks	↓ positive symptoms measured with PANSS, ↓ depressive symptoms
Emsley et al., 2002 [54]	ethyl-EPA 3 g/day + antipsychotics	Placebo + antipsychotics	40 patients with stable schizophrenia	12 weeks	↓ positive symptoms and negative symptoms measured with PANSS
Jamilian et al., 2014 [55]	ethyl-EPA 1 g/day + antipsychotics	Placebo + antipsychotics	60 patients with stable schizophrenia	8 weeks	↓ psychotic symptoms measured with PANSS
Bentsen et al., 2013 [56]	ethyl-EPA 2 g/day + antipsychotics	Placebo + antipsychotics	99 patients with stable schizophrenia	16 weeks	↓ impairment of the course of psychosis
Robinson et al., 2019 [57]	EPA 740 mg and DHA 400 mg/day + antipsychotics	Placebo+ Antipsychotics	50 patients with stable schizophrenia	16 weeks	↓ confusion, anxiety, depression, irritability and tiredness/fatigue
Emsley et al., 2006 [59]	ethyl-EPA 2 g/day + antipsychotics	Placebo + antipsychotics	77 patients with stable schizophrenia	12 weeks	no efficacy on specific psychotic symptoms
Fenton et al., 2001 [60]	ethyl-EPA 3 g/day + antipsychotics	Placebo + antipsychotics	87 patients with stable schizophrenia	16 weeks	no significant differences in positive, negative symptoms, mood or cognition

**Table 2:** Randomized controlled trials and open trials of omega-3 PUFAs on affective symptoms.

Study (Year) [Ref.]	Interventional Arm(s)	Comparison Arm(s)	Sample	Treatment Duration	Results
Pawelzcyk et al., 2018 [51]	EPA + DHA 2.2 g/day	placebo	71 patients at FEP	26 weeks	↑ level of telomerase in peripheral blood cells with ↓ depressive symptoms
Llorente et al., 2003 [79]	DHA 0.2 g/day monotherapy	Placebo	99 healthy pregnant women	16 weeks	no effect on postpartum depression
Marangell et al., 2003 [80]	add on to standard therapy DHA 2 g/day monotherapy	Standard therapy	36 patients with MDD	12 weeks	no significant differences
Silvers et al., 2005 [81]	EPA 0.6 g/day + DHA 2.4 g/day added to standard therapy	Standard therapy	77 patients with MDD	12 weeks	no evidence that n-3PUFAs improved mood compared to placebo. Mood improved in both groups within the first 2 weeks of the study
Greyner et al., 2007 [82]	EPA 0.6 g/day + DHA 2.2 g/day add to standard therapy	Standard therapy	83 patients with MDD	16 weeks	no significant differences
Hallahan et al., 2007 [134]	EPA (1.2 g/day) + DHA (0.9 g/day)	Placebo	49 patients with self-harm behaviors (35 BPD)	12 weeks	Improvement of depression, suicidality and reaction to daily stress
Freeman et al., 2008 [83]	EPA 1.1 g/day + DHA 0.8 g/day	placebo	59 women	8 weeks	no benefit on perinatal depressive symptoms
Jazayeri et al., 2008 [84]	EPA 1 g/day	fluoxetine 20 mg/day	60 patients with MDD	8 weeks	↓ depressive symptoms in both groups
Rees et al., 2008 [85]	ethyl-EPA 0.4 g/day + DHA 1.6 g/day	placebo	26 pregnant patients	6 weeks	no benefits on depressive symptoms
Rogers et al., 2008 [86]	EPA 0.63 g/day + DHA 0.85 g/day monotherapy	placebo	218 mild to moderate depressed patients untreated	12 weeks	n-3PUFAs not have beneficial or harmful effects on mood in mild to moderate depression.
Doornbos et al., 2009 [87]	DHA 0.22 g/day or DHA 0.22 g/day + AA (0.22 g/day arachidonic acid) monotherapy	placebo	119 healthy pregnant women	28 weeks	red blood cell DHA, AA and DHA/AA ratio did not correlate with EPDS or blues scores
Lucas et al., 2009 [88]	EPA 1.05 g/day + DHA 0.25 g/day mono-therapy	placebo	120 patients with psychological distress with or without MDD in comorbidity	8 weeks	no significant differences
Makrides et al., 2010 [89]	DHA-rich tuna oil capsules 0.5 g/day monotherapy	placebo	2399 healthy pregnant women at 21 weeks’ gestation	women received assigned capsules daily, from study entry until birth of their child	DHA during pregnancy did not lower levels of postpartum depression
Antypa et al., 2012 [90]	EPA 1.74 g/day+ DHA 0.25 g/day added to standard therapy	Standard therapy	71 patients with history of at least one MDD	4 weeks	no significant effects on memory, attention, cognitive reactivity and depressive symptoms
Mozurkewich et al., 2013 [91]	EPA 1.06 g/day+ DHA 0.27 g/day monotherapy	EPA 0.18 g/day + DHA 0.9 g/day monotherapy	126 healthy pregnant women	6–8 weeks	no differences between groups in BDI scores or other depression endpoints
Mischoulon et al., 2009 [92]	EPA 1 g/day + (+ 0.2% dL alphatocopherol) monotherapy	placebo	57 patients with MDD	8 weeks	↓ depressive symptoms assessed with HDRS, but no statistical significance
Park et al., 2015 [93]	EPA 1140 g/day + DHA 0.6 g/day add to standard therapy	Standard therapy	35 patients with MDD	12 weeks	no significant differences
Young et al., 2017 [94]	PEP + EPA 1.4 g/day + DHA 0.2 g/day + 0.4 g/day other	placebo	72 depressed patients 7–14 years old	12 weeks	↓ co-occurring behavior symptoms in youth with depression.
Gabbay et al., 2018 [95]	2:1 ratio of EPA to DHA: Initial dose of 1.2 g/day. Doses were raised in increments of 0.6 g/day every 2 weeks (maximum possible dose of 3.6 g/day, combined EPA 2.4 g + DHA 1.2 g)	placebo	51 psychotropic medication-free adolescents with MDD aged 12–19 years old	10 weeks	n-3PUFAs do not appear to be superior to placebo.
Tayama et al., 2019 [96]	DHA 500 mg/day + EPA 1000 mg/day	placebo	20 patients with mild-to-moderate depression	12 weeks	no significant differences
Nemets et al., 2006 [97]	ethyl-EPA 0.4 g/day + DHA 0.2 g/day	placebo	20 patients 6–12 years-old	16 weeks	↓ depressive symptoms measured with CDRS, CDI and CGI
Peet & Horrobin 2002 [98]	Ethyl-EPA 1/2/4 g/day + Standard therapy	Placebo + Standard therapy	70 patients with persistent depression despite ongoing treatment	12 weeks	Ethyl-EPA 1 g/day group > placebo group no significant differences in the Ethyl-EPA 2 and 4 g/day groups
Su et al., 2003 [99]	ethyl-EPA 4.4 g/day + DHA 2.2 g/day add-on existing antidepressant treatment	Placebo + existing antidepressant treatment	22 patients with MDD	8 weeks	↓ depressive symptoms measured with HDRS
Nemets et al., 2006 [100]	ethyl-EPA 0.4 g/day + DHA 0.2 g/day	Placebo	20 depressed patients 6–12 years-old	16 weeks	↓ depressive symptoms measured with CDRS, CDI and CGI
Mischoulon et al. (2009) [101]	EPA 1 g/day + (+0.2% dL alphatocopherol) monotherapy	Placebo	57 MDD patients	8 weeks	↓ depressive symptoms assessed with HDRS, but no statistical significance
Rondanelli et al., 2010,2011 [102,103]	EPA 1.67 g/day + DHA 0.83 g/day added to existing antidepressant treatment	Placebo + existing antidepressant treatment	46 elderly female residents in a nursing home	8 weeks	↓ depressive symptoms assessed with GDS, improvement of phospholipids fatty acids profile
Lespérance et al., 2011 [104]	EPA 1.05 g/day + DHA 0.15 g/day + existing antidepressant treatment	Placebo + existing antidepressant treatment	432 patients with a major depressive episode	8 weeks	↓ depressive symptoms only for patients without comorbid anxiety disorders
Tajalizadekhoob et al., 2011 [105]	EPA 0.18 g/day + DHA 0.12 g/day add to standard therapy (55 patients) or in monotherapy (11 patients)	Placebo	66 patients with mild-to moderate depression aged > 66 years	24 weeks	low-dose n-3PUFAs have some efficacy in mild to moderate depression
Gertsik et al., 2012 [106]	EPA 0.9 g/day + DHA 0.2 g/day + other n-3 PUFAs (0.1 g/day) added to citalopram	Placebo added to citalopram	42 MDD patients taking citalopram	9 weeks	significantly greater improvement in HDRS scores
Krawczyk et al., 2012 [107]	EPA 2.2 g/day + DHA 0.7 g/day + GLA (0.24 g/day) + vit. E added to standard therapy	Standard therapy	21 patients with severe episode of treatment resistant recurring depression	8 weeks	n-3PUFAs significantly improved HDRS scores
Rizzo et al., 2012 [108]	EPA/DHA 2.1/2.5 g of n3-PUFA monotherapy	Placebo	46 MMD patients (only women > 66 years old)	8 weeks	mean GDS score and AA/EPA ratio, in whole blood and RBC membrane phospholipids, were significantly lower
Mozaffari-Khosravi et al., 2012 [109]	EPA 1 g/day or DHA 1 g/day added to standard therapy	Placebo + Standard therapy	81 mild to moderate depressed patients	12 weeks	↓ HDRS score EPA > compared with those in the DHA or placebo groups
Judge et al., 2014 [110]	DHA 0.3 g/day	Placebo	42 healthy pregnant women	8 weeks	↓ depressive symptoms assessed with PDSS
Ginty et al., 2015 [111]	EPA + DHA 1.4 g/day monotherapy	Placebo	23 depressed patients	3 weeks	n-3PUFAs group had a significant reduction in BDI scores over time
Jahangard et al., 2018 [112]	n-3 PUFAs (1000 mg/day) + sertraline (50–200 mg/day)	sertraline (50–200 mg/day)	50 MDD outpatients	12 weeks	↓ depression, anxiety, sleep and patients’ competencies to regulate their emotions.
Chiu et al., 2005 [113]	4.4 g/day EPA + 2.4 g/day DHA added on valproate 2 g/day	valproate 2 g/day and	16 newly hospitalized patients in the acute manic phase of bipolar disorder	4 weeks	No significant differences
Hirashima et al., 2004 [115]	High dose: EPA, 5.0–5.2 g/day; DHA, 3.0–3.4 g/day; other, 0.3–1.7 g/day + standard therapy	Standard therapy	21 patients with bipolar disorder	4 weeks	No significant differences
Keck et al., 2006 [116]	EPA 6 g/day in addition to at least one mood stabilizer	at least one mood stabilizer	121 patients with bipolar depression or rapid cycling bipolar disorder	4 months	No significant differences
Frangou et al., 2006 [117]	ethyl-EPA 1 or 2 g/day added to stable psychotropic medications	stable psychotropic medications	75 patients with bipolar disorder	12 weeks	↓ depressive symptoms measured with HDRS
Murphy et al., 2012 [118]	omega-3 fatty acids plus cytidine or omega-3 fatty acid plus placebo in addition to a mood stabilizer	only placebo in addition to a mood stabilizer	45 patients with type I bipolar disorder	4 months	no benefits of omega-3 fatty acids on affective symptoms
Stoll et al., 1999 [119]	EPA 6.2 g/day + DHA 3.4 g/day	Placebo	30 patients with bipolar disorder	16 weeks	↓ depressive symptoms measured with HDRS
Gracious et al., 2010 [120]	ALA in addition to psychotropic medication	Standard therapy	children and adolescent with bipolar I or II disorder	16 weeks	significant improvement of overall symptom severity compared with placebo
Zanarini & Franknburg, 2003 [133]	Ethyl-EPA 1 g/day (with no standard psychiatric therapies)	placebo	30 females with BPD	8 weeks	↓ depression
Buydens-Branchey et al., 2006, 2008 [130,131]	eicosapentaenoic acid + docosahexaenoic acid 3 g/day	Placebo	44 patients with anxiety disorder and substance abuse disorder	3 months + 3 months after therapy discontinuation	↓ anxiety symptoms > in PUFAs group than in placebo one, also after therapy discontinuation

**Table 3:** Randomized controlled trials and open trials of omega-3 PUFAs on impulsivity and aggression.

Study (Year) [Ref.]	Interventional Arm(s)	Comparison Arm(s)	Sample	Treatment Duration	Results
Qiao et al., 2018 [61]	DHA 360 mg/day + EPA 540 mg/day + antipsychotics	Placebo + antipsychotics	50 patients with stable schizophrenia	12 weeks	↓ violence, but no improvement in positive and negative symptoms
Zanarini & Franknburg, 2003 [133]	Ethyl-EPA 1 g/day (with no standard psychiatric therapies)	placebo	30 females with BPD	8 weeks	↓ aggression
Ginty et al., 2017 [135]	EPA 1 g/day + DHA 0.4 g/day	Placebo	272 healthy volunteers	18 weeks	No significative differences on impulsive behaviors nor on corticolimbic and corticostriatal brain functionality
Bègue et al., 2017 [150]	EPA 0.772 g/day + DHA 0.638 g/day	Placebo	194 Participants aged 18–45 from the general population	6 weeks	Omega-3 supplements significantly decreased self-reported aggressiveness
Sinn and Bryan, 2008 [151]	EPA 93 mg/day + DHA 29 mg/day + gamma-linolenic acid 10 mg/day versus placebo. No ADHD medications	Placebo	132 children (7 to 12 years) with ADHD	15 weeks	improved in inattention, hyperactivity and impulsivity in most ADHD scales in parents reports; no improvement in teachers reports Limits: No ADHD diagnosis (reported ADHD symptoms)
Perera et al., 2012 [152]	omega-3 + omega-6 with ADHD medications	Placebo with ADHD medications	98 children (6 to 12 years) with ADHD diagnosis	6 months	improved behavior and learning in restlessness, aggressiveness, completing work and academic performance, but not in inattention, impulsiveness and cooperation with parents and teachers
Bellino et al., 2014 [153]	EPA (1.2 g/day) + DHA (0.8 g/day) + valproate (800–1300 mg/day)	Valproate (800–1300 mg/day)	43 BPD outpatients	12 weeks	No differences with regard to global symptoms. Improvement of impulsivity, anger and self-mutilating conducts in omega-3 group
Bozzatello et al., 2018 [154]	EPA (1.2 g/day) + DHA (0.8 g/day) + valproate (800–1300 mg/day)	Valproate (800–1300 mg/day)	43 BPD outpatients	24 weeks follow-up	Combined therapy with omega-3 fatty acids showed long-lasting effects after discontinuation in terms of anger control.

**Table 4:** Randomized controlled trials and open trials of omega-3 PUFAs on self-harm behaviors.

Study (Year) [Ref.]	Interventional Arm(s)	Comparison Arm(s)	Sample	Treatment Duration	Results
Hallahan et al., 2007 [134]	EPA (1.2 g/day) + DHA (0.9 g/day)	Placebo	49 patients with self-harm behaviors (35 BPD)	12 weeks	Improvement of self-harm behaviors, suicidality, and reaction to daily stress
Bellino et al., 2014 [153]	EPA (1.2 g/day) + DHA (0.8 g/day) + valproate (800–1300 mg/day)	Valproate (800–1300 mg/day)	43 BPD outpatients	12 weeks	No differences with regard to global symptoms. Improvement of impulsivity, anger and self-mutilating conducts in omega-3 group
Marriott et al., 2016 [160]	3300 mg of n-3 PUFAs/day with 1650 mg EPA plus 1650 mg DHA per day	Placebo	40 United States (U.S.) military Veterans and non-Veterans ages 18–90 years with suicide risk	6 months	↓ suicidal behaviors
Gallagher et al., 2017 [165]	n-3 PUFAs including EPA or DHA	Placebo	40 individuals who self-harmed and 40 controls	10 years	↓ self-harm, ↓depressive symptoms and ↓ impulsivity

## Abbreviations

AMP	Adenosine monophosphate
EPA	Eicosapentaenoic acid;
DHA	Docosahexaenoic acid;
ethyl-EPA	Ethyl-eicosapentaenoic acid;
AA	Arachidonic acid;
α-LA	α-lipoic acid;
UHR	Ultra-high risk;
FEP	First episode of psychosis;
CDRS	Childhood Depression Rating Scale;
CDI	Childhood Depression Inventory;
EPDS	Edinburgh Postnatal Depression Scale;
GDS	Geriatric Depression Scale;
↓	Decrease of;
↑	Increase of;
MDD	Major depressive disorder;
HDRS	Hamilton Depression Rating Scale;
BDI	Beck Depression Inventory;
PDSS	Postpartum Depression Screening Scale;
PANSSPEP	Positive and Negative Syndrome ScaleIndividual-Family Psychoeducational Psychotherapy;
BPD	Borderline personality disorder;
PTSD	Posttraumatic Stress Disorder;
PUFAs	Polyunsaturated fatty acid;
>	Superior of;
<	Inferior of;
ADHD	Attention Deficit Hyperactivity Disorder;
BDNF	Brain-Derived Neurotrophic Factor
